# Associations of Collegiate Football Career and Incident Concussion with Players' Health: A Longitudinal Study from the CARE Consortium

**DOI:** 10.1007/s40279-025-02234-1

**Published:** 2025-05-01

**Authors:** Katherine J. Hunzinger, Jaclyn B. Caccese, Connor A. Law, Rachael M. Wittmer, Thomas A. Buckley, Steven P. Broglio, Thomas W. McAllister, Michael A. McCrea, Paul F. Pasquina, Andrea L. C. Schneider, the CARE Consortium Investigators, Holly J. Benjamin, Christopher D’Lauro, James T. Eckner, Christopher C. Giza, Kevin M. Guskiewicz, Thomas W. Kaminski, Laura J. Lintner, Christina L Master, Jane McDevitt, Jason P. Mihalik, Chris Miles, Justus Ortega, Nicholas L. Port, Margot Putukian, Adam Susmarski

**Affiliations:** 1 Department of Exercise Science, Ronson Health and Applied Science Center, Thomas Jefferson University, 225E, 4201 Henry Ave., Philadelphia, PA 19144 USA; 2 Jefferson Center for Injury Research and Prevention, Thomas Jefferson University, Philadelphia, PA USA; 3 The Ohio State University Chronic Brain Injury Program, The Ohio State University College of Medicine, Columbus, OH USA; 4 Department of Neurology, University of Pennsylvania-Perelman School of Medicine, Philadelphia, PA USA; 5 Sidney Kimmel Medical College, Thomas Jefferson University, Philadelphia, PA USA; 6 Department of Kinesiology and Applied Physiology, University of Delaware, Newark, DE USA; 7 Interdisciplinary Program in Biomechanics and Movement Science, University of Delaware, Newark, DE USA; 8 Michigan Concussion Center, University of Michigan, Ann Arbor, MI USA; 9 Department of Psychiatry, Indiana University School of Medicine, Indianapolis, IN USA; 10 Department of Neurosurgery, Medical College of Wisconsin, Milwaukee, WI USA; 11 Walter Reed National Military Medical Center, Uniformed Services University of the Health Sciences, Bethesda, MD USA; 12 Department of Biostatistics, Epidemiology, and Informatics, University of Pennsylvania-Perelman School of Medicine, Philadelphia, PA USA

## Abstract

**Background:**

The influence of repetitive head impacts on collegiate football players remains unclear as prior research is often limited to small samples or short-term studies focused on single seasons.

**Objective:**

Our objective was to determine the associations between collegiate football career or incident concussion and changes in neurocognitive function, postural stability, and physical and psychological health.

**Methods:**

In total, 574 football players enrolled in the Grand Alliance Concussion Assessment, Research and Education (CARE) Consortium (median age 18.0 years [interquartile range 18.0–19.0], 52% white race, 26% with incident concussion) completed baseline and exit evaluations (i.e., beginning and end of collegiate career) consisting of neurocognitive, postural stability, and physical/psychological health assessments, specifically, Immediate Post-Concussion Assessment and Cognitive Testing, Standardized Assessment of Concussion, Balance Error Scoring System, Sport Concussion Assessment Tool-5 (SCAT-5) Symptom checklist, and the Brief Symptom Inventory-18. Adjusted linear regression models incorporating inverse probability of attrition weighting were used to compare changes in scores between baseline and exit evaluations overall and by incident concussion status.

**Results:**

Overall, athletes had small improvements in neurocognitive functioning and postural stability over time but had small increases in symptom severity. Both the incident concussion and no incident concussion groups improved similarly on neurocognitive and postural stability measures (all *p* > 0.05 for difference in change over time between incident concussion groups). Individuals with incident concussion reported fewer symptoms and lower symptom severity over time than did those without incident concussion (SCAT symptom count difference − 1.22; 95% confidence interval [CI] − 1.89 to − 0.54; SCAT symptom severity difference: − 2.46; 95% CI − 4.06 to − 0.86; Brief Symptom Inventory-18 somatization difference: − 0.55; 95% CI − 0.93 to − 0.17).

**Conclusions:**

Overall, collegiate football players demonstrated small, non-clinically meaningful improvements in neurocognitive function and postural stability. Moreover, athletes who experienced a concussion reported slight improvements in physical/psychological health symptoms over their collegiate careers.

**Supplementary Information:**

The online version contains supplementary material available at 10.1007/s40279-025-02234-1.

## Key Points

**Table:** 

Chronic exposure to sport-related concussion and repetitive head impacts through American football have been associated with subsequent dysfunction, but results have been mixed, and studies have been limited to short-term investigations and small sample sizes.
In a sample of over 500 American collegiate football players, there were no clinically meaningful negative changes in concussion assessment battery outcomes following a median of 3.3 years of collegiate football exposure or from incident concussion.
These findings suggest that multiple seasons of repetitive head impacts and proper concussion protocols may not lead to significant performance declines in football players. However, a need exists to follow these athletes into mid- to late-adulthood to understand the more chronic effects.

## Introduction

The long-term sequelae of sport-related concussions and repetitive head impacts (RHIs) have come under increasing investigation over the past two decades, particularly in the context of American football [1, 2]. The collision nature of the sport exposes athletes to a high number of RHIs, which have been associated with long-term morbidity and executive dysfunction when compared with other contact and non-contact sports [3]. Individuals who played tackle football at the collegiate level have been shown to be at increased risk of impaired executive function, memory loss, recurrent headaches, and behavior changes [4, 5]. Several studies have suggested that, decades after collegiate athletic careers, these individuals have an estimated two to four times higher prevalence of cognitive impairment [4, 5]. In contrast, other data have suggested that most former collegiate football players have longer, healthier lives and lower mortality than the general population [4, 6], with a systematic review from the 6th Consensus on Concussion in Sport indicating that only a small proportion of former professional athletes have greater neurologic dysfunction in later life than their peers [7].

Research on the associations of collegiate football, RHIs, and concussions with player health have been largely limited to single-season timelines and/or to only one team or smaller samples [8–15]. These studies have reported heterogeneous results, with some reporting no clinically meaningful changes on measures of neurocognitive function, gait, and balance [12, 14], others reporting improvements in verbal memory and reaction time [13], and others reporting negative changes in learning, balance, and spatial memory [8, 10, 11] across a single season. Despite many of these studies adjusting for concussion history, it is unknown how incident concussion during the study observation period may have affected results [16, 17]. Given the long-term sequelae of RHIs and concussion history on collision sport athlete health, it is theorized that athletes with incident concussion would perform worse over time than their peers without incident concussion [18–20]. Mixed findings, paired with limited research spanning multiple collegiate seasons highlights the need for further investigation on the multi-season effects of RHI exposure to collegiate football on player health.

The Concussion Assessment, Research and Education (CARE) Consortium is the largest prospective study on the natural history of sports-related concussion to date and is funded by the National Collegiate Athletics Association (NCAA) and Department of Defense (DOD). Leveraging data from this consortium, we can better understand the associations with a collegiate football career (with and without incident concussion events) in a diverse sample of division I, II, and III collegiate athletes [17, 21]. Our primary objective was to investigate changes in concussion assessment battery outcomes of cognitive function, postural stability, physical symptoms, and psychological health from initial (i.e., start of collegiate career at a CARE site) to exit (i.e., within 1 year of the end of collegiate career) assessments among collegiate football players. Our secondary objective was to evaluate potential differences in performance on concussion assessment battery outcomes over time among football players with versus without an incident concussion (i.e., incident during their collegiate career). We hypothesized that there would be no clinically meaningful differences in concussion assessment outcomes between initial (i.e., CARE site baseline) and exit assessments in the overall population and that individuals with an incident concussion would have worse performance and greater symptoms over time than individuals without incident concussion.

## Methods

### Participants

**Fig. 1:**
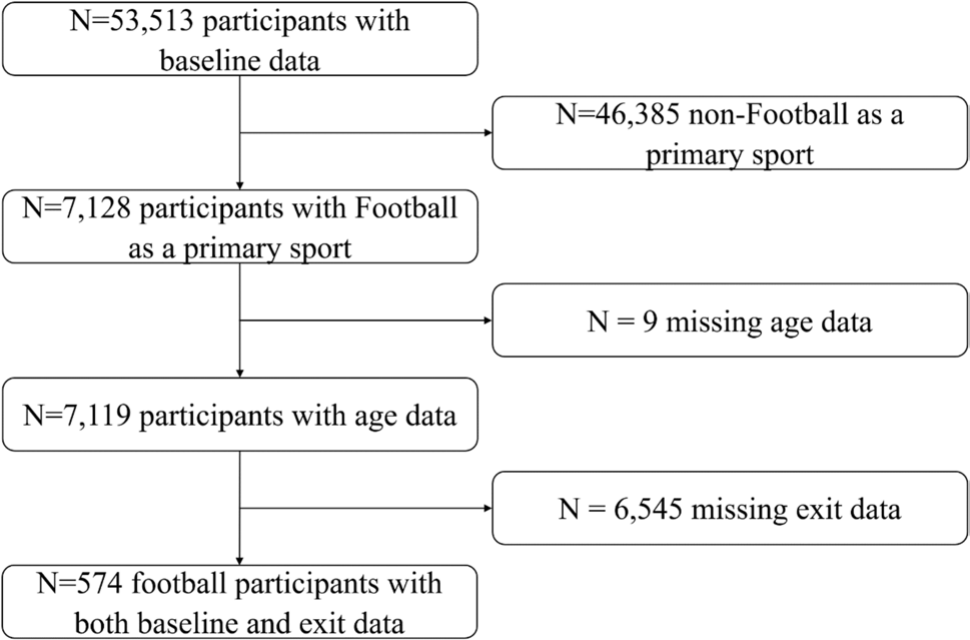
Inclusion and exclusion flow chart

The NCAA DOD CARE Consortium consists of 30 universities, including four military service academies [21]. The CARE Consortium enrolled and obtained initial baseline assessments from 53,513 athletes and cadets between August 2014 and May 2018 [17, 21]. For this study, we included all football players enrolled in the CARE Consortium who completed both an initial baseline and exit assessments [21]. Of 34,709 consented participants in the CARE Consortium with initial baseline data (i.e., assessment performed upon becoming a student athlete at a CARE Consortium site), 7199 football players (20.7%) had non-missing age data (*n* = 9 with missing age data). Of these, 574 (8.0%) completed at least one assessment during both initial and exit assessments (Fig. 1). Of note, exit assessments were heavily disrupted by the response to the coronavirus disease 2019 (COVID-19) pandemic, resulting in limited data. All included participants completed informed consent, and the research protocol was approved by each university’s institutional review board (IRB) and the US DOD Human Research Protections Office.

### Initial Baseline and Exit Concussion Assessment Battery

Initial baseline assessments took place at enrollment into the CARE Consortium (i.e., upon becoming a student athlete at a CARE Consortium site). Exit assessments took place within 1 year of the end of the individual’s collegiate career (defined by eligibility). During the initial baseline assessment, participants self-reported demographics and other variables, including history of diagnosed concussion and contact/collision sport history. Both baseline and exit assessments consisted of a standardized concussion assessment battery that was scored and assessed by trained staff members and has been described in detail elsewhere [21]. Consistent with concussion evaluation recommendations at the time of the study, assessments included measures of cognitive function (Immediate Post-Concussion Assessment and Cognitive Testing [ImPACT], Standardized Assessment of Concussion), postural stability (Balance Error Scoring System [BESS]), physical symptoms, and psychological health (Sport Concussion Assessment Tool [SCAT]–versions 3 and 5, Brief Symptom Inventory [BSI]-18) [21–23]. Details on each measure and the minimal clinically meaningful changes for each measure are provided in Table S1 in the electronic supplementary material (ESM).

### Incident Concussion

Data on incident diagnosed concussion (i.e., any diagnosed concussion occurring between initial baseline and exit assessments) were obtained from post-injury assessments. Participants were categorized into two groups: incident concussion or no incident concussion. Concussions were identified, assessed, and diagnosed by research and medical team members at each CARE site using a standardized definition from evidence-based guidelines [21, 24].

### Covariates

Statistical models were adjusted for the following covariates that were collected at initial baseline: years of contact sports (continuous), race (Black, other, white, missing), history of concussion at baseline (yes, no, missing), primary position played (speed, non-speed, special teams, missing), and time between baseline and exit assessments (continuous, days). “Years of contact sports” was calculated as the sum of self-reported years played at baseline of contact sports involving RHIs (i.e., boxing, football, ice hockey, lacrosse, rugby, soccer, and wrestling) [25]. Primary position played was categorized as speed (quarterback, running back, halfback, fullback, wide receiver, tight end, defensive back, safety, and linebacker), non-speed (all defensive and offensive linemen), or special teams [26].

### Statistical Analysis

Participant characteristics and concussion assessment battery scores are shown overall and stratified by incident concussion status as means and standard deviations or medians and interquartile range (25th–75th percentiles) and minimum–maximum for continuous data and as numbers and percentages for categorical data. Adjusted linear regression models were utilized to estimate change in concussion assessment battery scores over time; covariates included factors that may confound associations of collegiate football career and incident concussion with changes in neurocognitive function, postural stability, and physical and psychological health. Specifically, we adjusted for years of contact/collision sports, race, previous concussion history at initial baseline, primary position group, and days between initial baseline and exit assessments [25, 27, 28]. To standardize and compare effect sizes in the change from initial baseline to exit assessment between groups, z-scores for each test were calculated by subtracting the baseline population mean and dividing by the baseline population standard deviation. Z-scores for SCAT Symptom Severity; SCAT Symptoms; ImPACT Symptom Severity; ImPACT Reaction Time; BSI-18 Somatization; GSI Composite, Depression, and Anxiety; and BESS were inverted so that a positive z-score indicates the incident concussion group having demonstrated greater improvement from baseline to exit than the no concussion group for all tests. To address differential participation in exit assessments, our linear regression models incorporated stabilized inverse probability of attrition weights that were computed using a logistic regression model for participation versus non-participation in exit assessments that was adjusted for age at baseline, race, ethnicity, prior concussion at baseline, primary position, and years of contact/collision sports. Therefore, reported estimates are representative of the entire CARE Consortium football population with initial baseline data. In sensitivity analyses, we also performed complete case analyses and adjusted for number of prior concussions (0, 1, 2+) instead of concussion history (yes, no).

All analyses were performed using R statistical software, version 4.2.2 (R Foundation for Statistical Computing, Vienna, Austria) using tidyverse (version 2.0.0), Table 1 (version 1.4.3), magrittr (version 2.0.3), twang (version 2.6), and survey (version 4.2-1) packages.

**Table 1:** Participant characteristics among (primary) football players with initial baseline and exit data

Characteristic	Overall (*N* = 574)	No incident concussion (*N* = 425)	Incident concussion (*N* = 149)
Age, years	18.0 (18.0–19.0)	18.0 (18.0–19.0)	18.0 (18.0–19.0)
Sex, male	574 (100.0)	425 (100.0)	149 (100.0)
Race			
Black	190 (33.1)	137 (32.2)	53 (35.6)
Other	70 (12.2)	52 (12.2)	18 (12.1)
White	299 (52.1)	224 (52.7)	75 (50.3)
Missing	15 (2.6)	12 (2.8)	3 (2.0)
Ethnicity			
Hispanic	34 (5.9)	27 (6.4)	7 (4.7)
Non-Hispanic	430 (74.9)	320 (75.3)	110 (73.8)
Missing	110 (19.2)	78 (18.4)	32 (21.5)
Days from baseline to exit	1217 (930–1366)	1216 (900–1366)	1238 (988–1359)
Previous concussion history			
Yes	185 (32.2)	132 (31.1)	53 (35.6)
No	373 (65.0)	280 (65.9)	93 (62.4)
Missing	16 (2.8)	13 (3.1)	3 (2.0)
Previous concussions			
0	389 (67.8)	293 (68.9)	96 (64.4)
1	146 (25.4)	108 (25.4)	38 (25.5)
2+	39 (6.8)	24 (5.6)	15 (10.1)
Missing	16 (2.8)	13 (3.1)	3 (2.0)
Incident concussions			
0	425 (74.0)	NA	NA
1	116 (20.2)		116 (77.9)
2	26 (4.5)		26 (17.4)
3	5 (0.9)		5 (3.4)
4	2 (0.3)		2 (1.3)
Player primary position			
Non-speed	169 (29.4)	117 (27.5)	52 (34.9)
Special teams	43 (7.5)	40 (9.4)	3 (2.0)
Speed	343 (59.8)	251 (59.1)	92 (61.7)
Missing	19 (3.3)	17 (4.0)	2 (1.3)
Years of football exposure	9.0 (7.0–12.0)	9.0 (7.0–12.0)	9.0 (7.0–12.0)
Missingness	18	14 (3.3)	4 (2.7)
Years of contact/collision sports exposure	15.0 (9.0–20.0)	15.0 (9.0–20.0)	14.0 (9.0–20.0)

Data are presented as median (interquartile range) or *n* (%) unless otherwise indicated

*NA* not applicable

### Ethical Considerations

All study procedures were reviewed and approved by the University of Michigan IRB, the US Army Medical Research and Material Command Human Research Protections Office, and the local IRB at each of the performance sites. The study was performed in accordance with the standards of ethics outlined in the Declaration of Helsinki. The Thomas Jefferson University IRB approved the secondary data analysis and data access for this study (iRISID-2023-2273) on August 7, 2023.

## Results

In this cohort of 574 collegiate American football players followed for a median of 3.33 years (1217 days; range 84–2146), 149 (26.0%) had an incident concussion during their collegiate football career. Among these athletes with incident concussion, 33 (22.1%) had two or more incident concussions during their collegiate football careers (Table 1). Characteristics of athletes who did versus did not complete exit assessments (Table S2 in the ESM) with and without incident concussion (Table S3 in the ESM) were similar.

Across most outcomes, the athletes with both initial baseline and exit assessments (overall and stratified by incident concussion status) were stable or improved between baseline and exit assessment (Table 2). In weighted adjusted analyses (Table 3), there were statistically significant improvements in ImPACT Visual Memory (4.54 [95% CI 1.67–7.41]), ImPACT Visual Motor (2.86 [95% CI 1.75–3.98]), and BESS total score (− 2.40 [95% CI − 3.62 to − 1.18]) between baseline and exit in the overall population. All other outcomes had no statistically significant changes over time in the overall population.

**Table 2:** Baseline and exit assessment scores by concussion group among (primary) football players with initial baseline and exit data

Tool	Overall (*N* = 574)		No incident concussion (*N* = 425)		Incident concussion (*N* = 149)	
	Baseline	Exit median	Baseline median	Exit median	Baseline median	Exit median
ImPACT verbal memory	89.0 (80.0–96.0) [42.0, 100.0]	92.0 (82.0–98.0) [22.0, 100.0]	88.0 (81.0–96.0) [42.0, 100.0]	92.0 (81.0–98.0) [47.0, 100.0]	90.0 (78.8–96.0) [48.0, 100.0]	92.0 (85.0–98.0) [22.0, 100.0]
ImPACT visual memory	81.5 (70.0–90.0) [33.0, 100.0]	84.0 (74.0–92.0) [31.0, 100.0]	82.0 (69.8–90.0) [33.0, 100.0]	83.0 (74.0–91.0) [31.0, 100.0]	80.0 (71.0–90.0) [36.0, 100.0]	84.5 (74.0–92.3) [33.0, 100.0]
ImPACT visual motor	41.2 (36.1–45.6) [15.5, 55.0]	44.1 (38.4–48.6) [20.5, 53.6]	41.7 (35.7–45.9) [15.5, 55.0]	43.9 (37.9–48.6) [20.5, 53.4]	40.1 (36.3–44.9) [24.3, 53.6]	44.4 (39.4–48.6) [21.6, 53.6]
ImPACT reaction time	0.59 (0.54–0.65) [0.42, 1.46]	0.59 (0.54–0.65) [0.33, 1.13]	0.59 (0.54–0.66) [0.42, 0.97]	0.59 (0.54–0.65) [0.45, 1.13]	0.59 (0.55–0.65) [0.46, 1.46]	0.58 (0.54–0.65) [0.33, 0.93]
BESS total score	13.0 (9.0–18.0) [2.0, 40.0]	12.0 (8.0–16.0) [0.0, 40.0]	13.0 (9.0–18.0) [2.0, 40.0]	12.0 (8.0–16.0) [0.0, 40.0]	14.0 (10.0–18.0) [4.0, 35.0]	11.0 (8.0–15.0) [1.0, 40.0]
SCAT number of symptoms	1.0 (0.00–3.00) [0.0, 18.0]	0.00 (0.00–3.00) [0.0, 22.0]	0.50 (0.00–2.00) [0.0, 18.0]	1.00 (0.00–3.00) [0.0, 22.0]	1.00 (0.00–3.00) [0.0, 18.0]	0.00 (0.00–2.00) [0.0, 21.0]
SCAT symptom severity	1.0 (0.0–4.0) [0.0, 45.0]	0.0 (0.0–4.0) [0.0, 82.0]	0.5 (0.0–3.0) [0.0, 45.0]	1.0 (0.0–5.0) [0.0, 88.0]	1.0 (0.0–6.0) [0.0, 45.0]	0.0 (0.0–2.0) [0.0, 67.0]
SAC total score	27.0 (26.0–29.0) [15.0, 30.0]	28.0 (26.0–29.0) [20.0, 30.0]	27.0 (26.0–28.0) [15.0, 30.0]	28.0 (26.0–29.0) [20.0, 30.0]	27.0 (26.0–29.0) [19.0, 30.0]	28.0 (27.0–29.0) [22.0, 30.0]
BSI-18 GSI composite	0.0 (0.0–2.0) [0.0, 30.0]	0.0 (0.0–2.0) [0.0, 46.0]	0.0 (0.0–2.0) [0.0, 25.0]	0.0 (0.0–2.0) [0.0, 46.0]	0.0 (0.0–3.0) [0.0, 30.0]	0.0 (0.0–2.0) [0.0, 28.0]
BSI-18 depression	0.0 (0.0–0.0) [0.0, 11.0]	0.0 (0.0–1.0) [0.0, 17.0]	0.0 (0.0–0.0) [0.0, 9.0]	0.0 (0.0–1.0) [0.0, 17.0]	0.0 (0.0–0.8) [0.0, 11.0]	0.0 (0.0–0.3) [0.0, 15.0]
BSI-18 anxiety	0.0 (0.0–0.0) [0.0, 12.0]	0.0 (0.0–0.0) [0.0, 14.0]	0.0 (0.0–0.0) [0.0, 9.0]	0.0 (0.0–0.0) [0.0, 14.0]	0.0 (0.0–1.0) [0.0, 12.0]	0.0 (0.0–0.0) [0.0, 13.0]
BSI-18 somatization	0.0 (0.0–0.0) [0.0, 12.0]	0.0 (0.0–0.0) [0.0, 15.0]	0.0 (0.0–0.0) [0.0, 9.0]	0.0 (0.0–0.0) [0.0, 15.0]	0.0 (0.0–1.0) [0.0, 12.0]	0.0 (0.0–0.0) [0.0, 8.0]

Data are presented as median (interquartile range) [minimum, maximum]

90 participants were missing baseline ImPACT Verbal, Visual, Visual Motor, and Reaction Time scores; 24 were missing baseline BESS scores; 10 were missing baseline SCAT scores; 18 were missing baseline SAC scores; and 20 were missing baseline BSI-18 scores. 219 participants were missing exit IMPACT Verbal and Reaction Time scores; 221 were missing IMPACT Visual scores; 220 were missing IMPACT Visual Motor scores; 185 were missing exit BESS scores; seven were missing exit SCAT scores; 127 were missing exit SAC scores; and nine were missing exit baseline BSI-18 scores

*BESS* Balance Error Scoring System, *BSI* Brief Symptom Inventory, *GSI* Global Severity Index, *ImPACT* Immediate Post-Concussion Assessment and Cognitive Testing, *SAC* Standardized Assessment of Concussion, *SCAT* Sport Concussion Assessment Tool

**Table 3:** Weighted adjusted change in concussion battery outcomes from initial baseline to exit assessment

Test	Change in overall population	Change in no incident concussion group	Change in incident concussion group	Difference in change from baseline to exit between those with and without incident concussion
ImPACT verbal memory	1.29 (− 0.99–3.57)	1.07 (− 1.39–3.53)	1.87 (− 1.43–5.18)	0.80 (− 2.52–4.12)
ImPACT visual memory	**4.54 (1.67–7.41)**	**3.74 (0.73–6.75)**	**6.60 (2.92–10.28)**	2.86 (− 0.33–6.06)
ImPACT visual motor	**2.86 (1.75–3.98)**	**2.78 (1.56–4.00)**	**3.09 (1.76–4.41)**	0.31 (− 0.93–1.55)
ImPACT reaction time	− 0.01 (− 0.03–0.01)	0.00 (− 0.02–0.02)	− 0.03 (− 0.05–0.00)	− 0.02 (− 0.05–0.00)
BESS total score	** − 2.40 (− 3.62 to − 1.18)**	** − 2.59 (− 3.93 to − 1.26)**	** − 1.89 (− 3.44 to − 0.35)**	0.70 (− 0.84–2.24)
SCAT number of symptoms	0.34 (− 0.25–0.92)	**0.66 (0.03–1.28)**	− 0.56 (− 1.26–0.14)	** − 1.22 (− 1.89 to − 0.54)**
SCAT symptom severity	0.57 (− 0.72–1.86)	1.21 (− 0.15–2.58)	− 1.25 (− 2.91–0.41)	** − 2.46 (− 4.06 to − 0.86)**
SAC total score	0.14 (− 0.27–0.56)	0.02 (− 0.41–0.44)	0.49 (− 0.12–1.10)	0.47 (− 0.06–1.01)
BSI-18 GSI composite	0.31 (− 0.38–1.01)	0.56 (− 0.22–1.33)	− 0.40 (− 1.33–0.52)	− 0.96 (− 1.95–0.02)
BSI-18 depression	0.18 (− 0.13–0.49)	0.23 (− 0.10–0.57)	0.02 (− 0.42–0.45)	− 0.22 (− 0.66–0.22)
BSI-18 anxiety	0.10 (− 0.18–0.37)	0.15 (− 0.15–0.44)	− 0.05 (− 0.43–0.33)	− 0.19 (− 0.56–0.18)
BSI-18 somatization	0.04 (− 0.24–0.32)	0.18 (− 0.13–0.49)	** − 0.37 (− 0.73 to − 0.01)**	** − 0.55 (− 0.93 to − 0.17)**

Data are presented as mean difference (95% confidence interval). Statistically significant changes are noted in bold. Model adjusted for years of contact sports at baseline, race, time between baseline and exit assessments, concussion at baseline, and primary position

*BESS* Balance Error Scoring System, *BSI* Brief Symptom Inventory, *GSI* Global Severity Index, *ImPACT* Immediate Post-Concussion Assessment and Cognitive Testing, *SAC* Standardized Assessment of Concussion, *SCAT* Sport Concussion Assessment Tool

**Fig. 2:**
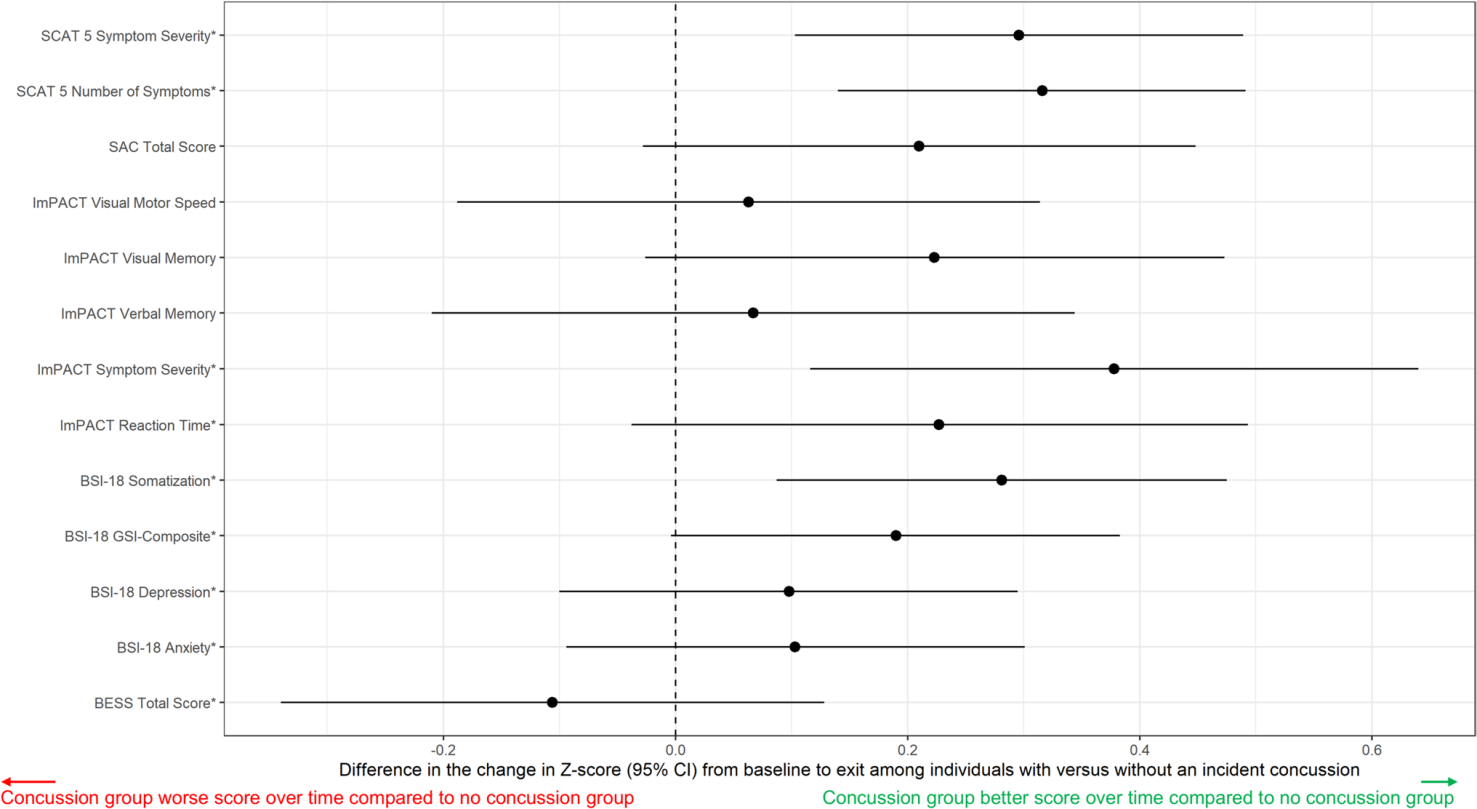
Changes in Z-scores by test. A positive z-score indicates that the incident concussion group demonstrated a greater improvement from baseline to exit than the no concussion group for all tests. Z-scores were inverted for the following variables, as denoted by an asterisk (*): Sport Concussion Assessment Tool (SCAT) Symptom Severity; SCAT Symptoms; Immediate Post-Concussion Assessment and Cognitive Testing (ImPACT) Symptom Severity; ImPACT Reaction Time; Brief Symptom Inventory (BSI)-18 Somatization; Global Severity Index (GSI) Composite, Depression, and Anxiety; and Balance Error Scoring System (BESS). *CI* confidence interval, *SAC* Standardized Assessment of Concussion

In weighted adjusted models, the incident concussion and no incident concussion group improved similarly between assessments on ImPACT Visual Memory, ImPACT Visual Motor, and BESS. Individuals with incident concussion reported fewer symptoms and lower symptom severity over time, whereas individuals without incident concussion reported more symptoms and higher symptom severity over time (SCAT Number of Symptoms difference − 1.22; 95% CI − 1.89 to − 0.54; SCAT Symptom Severity difference: − 2.46; 95% CI − 4.06 to − 0.86; and BSI-18 Somatization component score difference: − 0.55; 95% CI − 0.93 to − 0.17) (Table 3). The standardized z-scores for these changes are presented in Fig. 2. All observed changes over time were small and did not meet thresholds for clinically meaningful differences (Table S1 in the ESM). Results from complete case analyses (Table S4 in the ESM) were similar to those from the main analyses. When controlling for number of previous concussions at initial baseline (0, 1, 2+), difference scores between groups were similar to the primary analysis when controlling for concussion history (yes, no) (Table S5 in the ESM).

## Discussion

This study investigated associations between collegiate football careers or incident concussion and changes in concussion assessment battery measures over time. In this cohort of collegiate American football players followed for a median of 3.3 years across a collegiate football career, there were no negative changes in concussion assessment battery outcomes. Furthermore, the overall cohort showed minor improvements in some outcomes over time, with the incident concussion group having statistically significant greater improvements in measurements of concussion symptoms. However, it is important to note that these observed changes do not represent clinically meaningful difference values [29–31].

Overall, collegiate football players improved on concussion battery outcomes over time, with individuals with incident concussion demonstrating greater improvements on measures of symptoms over time than those without incident concussion. Improvements in concussion symptom outcomes are likely multifactorial and may be partly due to repeated test exposure (i.e., regression to the mean, where high symptom counts naturally tend to decrease on subsequent measurements), particularly in the incident concussion group, who were tested multiple times after injury. These assessments have been shown to have weak reliability and low clinical utility for most of these assessments outside the acute timeframe after concussion [29]. It is also possible that post-incident concussion rehabilitation measures and/or maturation contribute to these improvements [29–31]. This is in agreement with previous CARE Consortium data highlighting improvements in mean scores for ImPACT, Standardized Assessment of Concussion, BESS, and SCAT symptoms and severity across years 1, 2, and 3 among > 4800 collegiate student athletes, with authors noting small-to-medium learning effects [29, 32]. It is also important to consider that we were unable to determine the stability of testing conditions at baseline and exit assessments, whereby variability in testing environments (e.g., group or solo testing), participant traits (e.g., sleep levels, psychiatric disorders, anxiety), and timing of assessments (e.g., season), among other conditions, may have influenced test performance [33–35]. Although these findings reached statistical significance, reflecting improvements over time, these changes did not meet the criteria for clinically meaningful change [29, 32, 36].

A secondary explanation for the improvement in symptoms over time may be the result of the timing of baseline and exit assessments. CARE baseline assessments on football players were conducted at study enrollment, which was typically before an athlete’s first season at the university. Entering university for the first time can cause high levels of stress and anxiety, which can negatively affect sleep quality and performance on neurocognitive and physical/psychological assessments (e.g., ImPACT, BSI-18, symptom assessments) and may result in higher concussion-related symptom reporting among student athletes and cadets [33–35, 37]. Moreover, as noted in other CARE Consortium work, symptoms reported on the SCAT may or may not reflect an athlete’s baseline or usual symptom experience, depending on how they interpret the instructions at the time, which may further contribute to the observed changes over time [38]. Other work among collision sport athletes in the CARE Consortium has highlighted improvements over multiple seasons, evidenced by lower (i.e., better) BSI-18 scores and post-concussion symptoms scale symptom severity over three consecutive collegiate soccer seasons [32]. These findings among soccer players are consistent with our finding that multiple football seasons did not negatively affect symptoms or behavioral outcomes. Collectively, these data suggest that a collegiate football career’s worth of RHIs and incident concussion are not negatively associated with changes in cognitive, postural stability, or psychological health from initial baseline to exit assessments when assessed by common concussion clinical screen tools.

Certain limitations should be considered in the interpretation of our results. As noted above, the observed improvements over time are small, do not reach clinically meaningful difference thresholds, and may be the result of repeated assessment exposure and practice effects [29–31]. Yet, it remains unknown how many times these athletes were exposed to each test component over their entire sporting careers (i.e., collegiate and prior) or whether athletes in the no concussion group failed to disclose a concussion during study participation. Second, a substantial proportion of football players did not complete an exit assessment. This may be because it was not offered during the early years of CARE (< 2018) or because of the shifts to online-only university environments due to COVID-19 (starting in March 2020) at the end of their collegiate football career, resulting in a large amount of missingness. However, we observed no group differences among individuals who received exit assessments and those who did not, including across incident concussion groups. We employed inverse probability of attrition weighting to account for the differential participation in exit assessments in our main analyses. Another limitation is that stratified analyses were grouped by incident diagnosed concussion status; whether some individuals with unreported or undiagnosed concussions were assigned to the no incident concussion group remains unknown, and this would result in biasing results towards the null. Lastly, the effect of post-concussion rehabilitation programs on changes in concussion assessment battery outcomes is also unclear because we did not have information on which participants participated in rehabilitation programs or the specific protocols they engaged in. Therefore, more work is needed to determine whether improvements in these measures in the incident concussion group may be attributed to injury rehabilitation protocols.

## Conclusion

In this cohort of collegiate football players, we did not observe any clinically meaningful associations between collegiate football career or incident concussion and changes in common concussion assessment battery outcomes over multiple years. These findings highlight that collegiate football players do not exhibit negative changes in neurocognitive function, postural stability, or psychological health over an entire college career of football-related RHI exposure. The lack of long-term deficits in current collegiate football players who sustain a concussion may indicate that the current concussion management strategies are effective. Future research should aim to investigate longer follow-up periods with athletes and conduct inquiries into the impact of resiliency and rehabilitation on these outcomes over time.

## Supplementary Information

Below is the link to the electronic supplementary material.

Supplementary file1 (PDF 268 KB)

## Declarations

### Availability of data and material

Data used in this investigation are available in the Federal Interagency Traumatic Brain Injury Research Informatics System (https://fitbir.nih.gov/).
