# Supplementary material for: Associations of Collegiate Football Career and Incident Concussion with Players' Health: A Longitudinal Study from the CARE Consortium
Source: Sports Med. 2025 May 1;55(9):2313–22. doi: 10.1007/s40279-025-02234-1 (PMC12476394; doi:10.1007/s40279-025-02234-1)
Supplement: Supplementary file 1 — Supplementary file1 (PDF 268 KB) [file 40279_2025_2234_MOESM1_ESM.pdf]

Supplementary Material

Journal: *Sports Medicine*

Title: Associations of Collegiate Football Career and Incident Concussion with Players' Health: A Longitudinal Study from the CARE Consortium

Authors:

Katherine J. Hunzinger, PhD, ACSM-CEP[1,2], Jaclyn B. Caccese, PhD[3], Connor A. Law, BS[4], Rachael M. Wittmer, BS[5], Thomas A. Buckley, EdD, ATC[6,7], Steven P. Broglio, PhD[8], Thomas W. McAllister, MD[9], Michael A. McCrea, PhD, ABPP[10], Paul F. Pasquina, MD[11], Andrea L.C. Schneider, MD, PhD[4,12], and the CARE Consortium Investigators

Affiliations:

[1]: Department of Exercise Science, Thomas Jefferson University, Philadelphia, PA

[2]: Jefferson Center for Injury Research & Prevention, Thomas Jefferson University, Philadelphia, PA

[3]: The Ohio State University Chronic Brain Injury Program, The Ohio State University College of Medicine, Columbus, OH

[4]: Department of Neurology, University of Pennsylvania-Perelman School of Medicine, Philadelphia, PA

[5]: Sidney Kimmel Medical College, Thomas Jefferson University, Philadelphia PA

[6]: Department of Kinesiology and Applied Physiology, University of Delaware, Newark, DE

[7]: Interdisciplinary Program in Biomechanics and Movement Science, University of Delaware, Newark, DE

[8]: Michigan Concussion Center, University of Michigan, Ann Arbor, MI

[9]: Department of Psychiatry, Indiana University School of Medicine, Indianapolis, IN

[10]: Department of Neurosurgery, Medical College of Wisconsin, Milwaukee, WI

[11]: Walter Reed National Military Medical Center, Uniformed Services University of the Health Sciences, Bethesda, MD

[12]: Department of Biostatistics, Epidemiology, and Informatics, University of Pennsylvania-Perelman School of Medicine, Philadelphia, PA

Corresponding Author:

Katherine J. Hunzinger, PhD, CEP

Department of Exercise Science, Thomas Jefferson University

Address: 225E Ronson Health and Applied Science Center, 4201 Henry Ave., Philadelphia, PA 19144, USA

Telephone: (215) 951-0487

Email: Katherine.Hunzinger@Jefferson.edu

Twitter: @KatieHunzinger

ORCID iD: 0000-0002-4599-8543

Supplemental Table 1. Description of Concussion Assessment Battery Outcome Measures

	**Outcome Measures**	**Score Description**	**Interpretation**
**ImPACT**	ImPACT Verbal Memory	Evaluates attentional processes, memory, and learning	Higher indicates a better score. Minimal detectable change (MDC) of 14.19.[37]
	ImPACT Visual Memory	Evaluates visual learning, memory, attention, and scanning.	Higher indicates a better score. MDC of 17.25.[37]
	ImPACT Visual Motor Speed	Derived from visual learning and memory, visual-motor response speed, and visual processing.	Higher indicates a better score. MDC of 11.07.[37]
	ImPACT Reaction Time	Average response speed. (reported in seconds)	Higher score indicates a slower reaction speed. MDC of 0.17s.[37]
**BESS**	Total Score	Total error count across firm and foam surface conditions and all three positions (i.e., feet together, single leg, and tandem). (Range 0 – 60)	Higher scores indicated worse postural stability. MDC of 7.3 (intra-rater) or 9.4 (inter-rater) points.[39]
**SCAT 5**	Number of Symptoms	22 Symptoms are evaluated for presence of symptom (Range 0-22)	Higher scores indicate greater prevalence of symptoms. MDC of 2.38 points 45 days post-concussion.[40]
	Symptom Severity	Sum of symptom severities across all 22 symptoms rated 0 (none) to 6 (severe). (Range 0-132)	Higher scores indicate greater symptom severity.
**SAC**	Total Score	0 (none correct) – 30 (all correct)	Higher scores indicate greater cognitive functioning. MDC of 1.85 points.[41]
**BSI-18**	Depression	Sum of 6 depression themed questions answered 0 ("not at all") – 4 ("extremely"). (Range 0 – 24)	Higher scores indicate worse depression.
	Anxiety	Sum of 6 anxiety themed questions answered 0 ("not at all") – 4 ("extremely"). (Range 0 – 24)	Higher scores indicate worse anxiety.
	Somatization	Sum of 6 somatization themed questions answered 0 ("not at all") – 4 ("extremely"). (Range 0 – 24)	Higher scores indicate worse somatization.
	GSI-Composite	Sum of all 18 questions answered 0 ("not at all") – 4 ("extremely"). (Range 0 – 72).	Higher scores indicating higher levels of psychological stress.

Supplemental Table 2. Participant Characteristics among (Primary) Football Players with Baseline Data Stratified by the Presence or Absence of Exit Data.

	**Baseline Only (N = 6,545)**	**Baseline and Exit Data (N = 574)**
Age (years; median, 25%, 75%)	19.0 (18.0, 20.0)	18.0 (18.00, 19.0)
Sex (Male)	6,545 (100.0%)	574 (100.0%)
Race		
Black	2,360 (36.1%)	190 (33.1%)
Missing	208 (3.2%)	15 (2.6%)
Other	792 (12.1%)	70 (12.2%)
White	3,185 (48.7%)	299 (52.1%)
Ethnicity		
Hispanic	475 (7.3%)	34 (5.9%)
Non-Hispanic	4,575 (69.9%)	430 (74.9%)
Missing	1,495 (22.8%)	100 (19.2%)
Days from Baseline to Exit		
Median (25, 75%)	N/A	1,217 (930, 1,366)
Missingness	6,545 (100.0%)	0 (0.0%)
Previous Concussion History		
Yes	2,094 (32.0%)	185 (32.2%)
No	4,283 (65.4%)	373 (65.0%)
Missing	168 (2.6%)	16 (2.8%)
Number Previous Concussion		
0	4,452 (68.0%)	389 (67.8%)
1	1,614 (24.7%)	146 (25.4%)
2+	479 (7.3%)	39 (6.8%)
Player primary position		
Speed	4,119 (62.9%)	343 (59.8%)
Non-Speed	1,774 (27.1%)	169 (29.4%)
Special Teams	394 (6.0%)	43 (7.5%%)
Missing	258 (3.9%)	19 (3.3%)
Years of football exposure		
Median (25, 75%)	10.0 (7.0, 12.0)	9.0 (7.0, 12.0)
Missingness	222 (3.4%)	18 (3.1%)
Years of contact/collision sports exposure		
Median (25, 75%)	14.0 (10.0, 20.0)	15.0 (9.0, 20.0)
**Baseline Assessments**		
ImPACT Memory Composite - Verbal		
Median (25, 75%)	88.0 (79.0, 95.0)	89.0 (80.0, 96.0)
Missingness	1,314 (20.1%)	90 (15.7%)

ImPACT Memory Composite - Visual		
Median (25, 75%)	78.0 (69.0, 88.0)	81.5 (70.0, 90.0)
Missingness	1,317 (20.1%)	90 (15.7%)
ImPACT Visual Motor Speed		
Median (25, 75%)	39.7 (34.9, 44.7)	41.2 (36.1, 45.6)
Missingness	1,320 (20.2%)	90 (15.7%)
ImPACT Reaction Time Composite		
Median (25, 75%)	0.61 (0.56, 0.68)	0.59 (0.54, 0.65)
Missingness	1,315 (20.1%)	90 (15.7%)
BESS Total		
Median (25, 75%)	13.0 (10.0, 18.0)	13.0 (9.0, 18.0)
Missingness	238 (3.6%)	24 (4.2%)
SCAT 3 Number of symptoms		
Median (25, 75%)	1.0 (0.0, 3.0)	1.0 (0.0, 3.0)
Missingness	99 (1.5%)	10 (1.7%)
SCAT 3 Symptom Severity		
Median (25, 75%)	1.0 (0.0, 4.0)	1.0 (0.0, 4.0)
Missingness	99 (1.5%)	10 (1.7%)
SAC (Total)		
Median (25, 75%)	27.0 (25.0, 28.0)	27.0 (26.0, 29.0)
Missingness	161 (2.5%)	18 (3.1%)
BSI-18 GSI Composite		
Median (25, 75%)	0.0 (0.0, 2.0)	0.0 (0.0, 2.0)
Missingness	209 (3.2%)	20 (3.5%)
BSI-18 Depression Raw		
Median (25, 75%)	0.0 (0.0, 0.0)	0.0 (0.0, 0.0)
Missingness	209 (3.2%)	20 (3.5%)
BSI-18 Anxiety Raw		
Median (25, 75%)	0.0 (0.0, 0.0)	0.0 (0.0, 000)
Missingness	209 (3.2%)	20 (3.5%)
BSI-18 Somatization Raw		
Median (25, 75%)	0.0 (0.0, 0.0)	0.0 (0.0, 0.0)
Missingness	209 (3.2%)	20 (3.5%)

Supplemental Table 3. Participant Characteristics among (Primary) Football Players with Baseline Data Stratified by the Presence or Absence of Exit Data and by Incident Concussion Status.

	BL Only (N = 6,545)	BL and Exit No Concussion (N = 425)	BL and Exit Concussion (N =149)	Overall (N= 7,119)
Age (years; median, 25%, 75%)	19.0 (18.0, 20.0)	18.0 (18.0, 19.0)	18.0 (18.0, 19.0)	19.0 (18.0, 20.0)
Sex (Male)	6,545 (100.0%)	425 (100.0%)	149 (100.0%)	7,119 (100.0%)
Race				
Black	2,360 (36.1%)	137 (32.2%)	53 (35.6%)	2,550 (35.8%)
Other	792 (12.1%)	52 (12.2%)	18 (12.1%)	862 (12.1%)
White	3,185 (48.7%)	224 (52.7%)	75 (50.3%)	3,484 (48.9%)
Missing	208 (3.2%)	12 (2.8%)	3 (2.0%)	223 (3.1%)
Ethnicity				
Hispanic	424 (7.6%)	27 (6.4%)	7 (4.7%)	509 (7.1%)
Non-Hispanic	3,923 (70.0%)	320 (75.3%)	110 (73.8%)	5,005 (70.3%)
Missing	1,257 (22.4%)	78 (18.4%)	32 (21.5%)	1,605 (22.5%)
Days from Baseline to Exit				
Median (25%, 75%)	N/A	1,216 (900, 1,366)	1,238 (988, 1,359)	1,217 (930, 1,366)
Missingness	6,545 (100.0%)	0 (0.0%)	0 (0.0%)	6,545 (91.9%)
Previous Concussion History				
Yes	2,094 (32.0%)	132 (31.1%)	53 (35.6%)	2,279 (32.0%)
No	4,283 (65.4%)	280 (65.9%)	93 (62.4%)	4,656 (65.4%)
Missing	168 (2.6%)	13 (3.1%)	3 (2.0%)	184
Number Previous Concussion				
0	4,452 (68.0%)	293 (68.9%)	96 (64.4%)	4,841 (68.0%)
1	1,614 (24.7%)	108 (25.4%)	38 (25.5%)	1,760 (24.7%)
2+	479 (7.3%)	24 (5.6%)	15 (10.1%)	518 (7.3%)
Player primary position				
Speed	4,119 (62.9%)	251 (59.1%)	92 (61.7%)	4,462 (62.7%)
Non-Speed	1,774 (27.1%)	117 (27.5%)	52 (34.9%)	1,943 (27.3%)
Special Teams	394 (6.0%)	40 (9.4%)	3 (2.0%)	437 (6.1%)
Missing	258 (3.9%)	17 (4.0%)	2 (1.3%)	277 (3.9%)

Years of football exposure				
Median (25%, 75%)	10.0 (7.0, 12.0)	9.0 (7.0, 12.0)	9.0 (7.0, 12.0)	10.0 (7.0, 12.0)
Missingness	222 (3.4%)	14 (3.3%)	4 (2.7%)	240 (3.4%)
Years of contact/collision sports exposure				
Median (25%, 75%)	14.0 (10.0, 20.0)	15.0 (9.0, 20.0)	14.0 (9.0, 20.0)	14.0 (9.0, 20.0)
**Baseline Assessments**				
ImPACT Memory Composite - Verbal				
Median (25%, 75%)	88.0 (79.0, 95.0)	88.0 (81.0, 96.0)	90.0 (78.8, 96.0)	88.0 (79.0, 95.0)
Missingness	1,314 (20.1%)	73 (17.2%)	17 (11.4%)	1,404 (19.7%)
ImPACT Memory Composite - Visual				
Median (25%, 75%)	78.0 (69.0, 88.0)	82.0 (69.8, 90.0)	80.0 (71.0, 90.0)	79.0 (69.0, 88.0)
Missingness	1,317 (20.1%)	73 (17.2%)	17 (11.4%)	1,407 (19.8%)
ImPACT Visual Motor Speed				
Median (25%, 75%)	39.5 (34.9, 44.7)	41.7 (35.7, 45.9)	40.1 (36.3, 44.9)	39.8 (35.0, 44.8)
Missingness	1,320 (20.2%)	73 (17.2%)	17 (11.4%)	1,410 (19.8%)
ImPACT Reaction Time Composite				
Median (25%, 75%)	0.61 (0.56, 0.68)	0.59 (0.54, 0.60)	0.59 (0.55, 0.65)	0.61 (0.56, 0.67)
Missingness	1,315 (20.1%)	73 (17.2%)	17 (11.4%)	1,405 (19.7%)
BESS Total				
Median (25%, 75%)	13.0 (10.0, 18.0)	13.0 (9.0, 18.0)	14.0 (10.0, 18.0)	13.0 (10.0, 18.0)
Missingness	238 (3.6%)	16 (3.8%)	8 (5.4%)	262 (3.7%)
SCAT 3 Number of symptoms				
Median (25%, 75%)	1.0 (0.0, 3.0)	0.5 (0.0, 2.0)	1.0 (0.0, 3.0)	1.0 (0.0, 3.0)
Missingness	99 (1.5%)	9 (2.1%)	1 (0.7%)	109 (1.5%)
SCAT 3 Symptom Severity				
Median (25%, 75%)	1.0 (0.0, 4.0)	0.5 (0.0, 3.0)	1.0 (0.0, 6.0)	1.0 (0.0, 4.0)
Missingness	99 (1.5%)	9 (2.1%)	1 (0.7%)	109 (1.5%)
SAC (Total)				
Median (25%, 75%)	27.0 (25.0, 28.0)	27.0 (26.0, 28.0)	27.0 (26.0, 29.0)	27.0 (25.0, 28.0)
Missingness	161 (2.5%)	12 (2.8%)	6 (4.0%)	179 (2.5%)
BSI-18 GSI Composite				
Median (25%, 75%)	0.0 (0.0, 2.0)	0.0 (0.0, 2.0)	0.0 (0.0, 3.0)	0.0 (0.0, 2.0)

Missingness	209 (3.2%)	17 (4.0%)	3 (2.0%)	229 (3.2%)
BSI-18 Depression Raw				
Median (25%, 75%)	0.0 (0.0, 0.0)	0.0 (0.0, 0.0)	0.0 (0.0, 0.8)	0.0 (0.0, 0.0)
Missingness	209 (3.2%)	17 (4.0%)	3 (2.0%)	229 (3.2%)
BSI-18 Anxiety Raw				
Median (25%, 75%)	0.0 (0.0, 0.0)	0.0 (0.0, 0.0)	0.0 (0.0, 1.0)	0.0 (0.0, 0.0)
Missingness	209 (3.2%)	17 (4.0%)	3 (2.0%)	229 (3.2%)
BSI-18 Somatization Raw				
Median (25%, 75%)	0.0 (0.0, 0.0)	0.0 (0.0, 0.0)	0.0 (0.0, 1.0)	0.0 (0.0, 0.0)
Missingness	209 (3.2%)	17 (4.0%)	3 (2.0%)	229 (3.2%)

Supplemental Table 4. Complete Case Adjusted Change in Concussion Battery Outcomes from Baseline to Exit Evaluation.

	Change in Overall Population	Change in No Incident Concussion Group	Change in Incident Concussion Group	Difference in the Change in from Baseline to Exit Comparing Individuals with Versus without an Incident Concussion
ImPACT Verbal	1.50 (-0.90, 3.90)	1.33 (-1.21, 3.87)	1.95 (-1.26, 5.15)	0.61 (-2.31, 3.54)
ImPACT Visual	**4.76 (2.23, 7.29)**	**4.15 (1.48, 6.83)**	**6.32 (2.95, 9.69)**	2.17 (-0.93, 5.26)
ImPACT Visual Motor	**3.17 (2.19, 4.15)**	**3.01 (1.98, 4.05)**	**3.56 (2.26, 4.87)**	0.55 (-0.65, 1.75)
ImPACT Reaction Time	-0.01 (-0.03, 0.01)	0.00 (-0.03, 0.02)	-0.03 (-0.05, 0.00)	-0.02 (-0.05, 0.00)
BESS Total Score	**-2.34 (-3.57, -1.10)**	**-2.46 (-3.77, -1.16)**	**-2.00 (-3.65, -0.34)**	0.47 (-1.04, 1.98)
SCAT Number of Symptoms	0.36 (-0.26, 0.97)	**0.68 (0.05, 1.32)**	-0.57 (-1.38, 0.25)	**-1.25 (-1.98, -0.52)**
SCAT Symptom Severity	0.52 (-0.81, 1.85)	1.15 (-0.24, 2.53)	-1.25 (-3.02, 0.51)	**-2.40 (-3.99, -0.82)**
SAC Total Score	**0.21 (-0.20, 0.62)**	0.09 (-0.33, 0.52)	**0.53 (-0.01, 1.08)**	0.44 (-0.05, 0.93)
BSI-18 GSI Composite	0.17 (-0.65, 0.99)	0.39 (-0.47, 1.24)	-0.49 (-1.58, 0.61)	-0.87 (-1.86, 0.11)
BSI-18 Depression	0.12 (-0.25, 0.48)	0.16 (-0.22, 0.54)	-0.01 (-0.49, 0.48)	-0.16 (-0.60, 0.27)
BSI-18 Anxiety	0.02 (-0.28, 0.32)	0.08 (-0.24, 0.39)	-0.15 (-0.56, 0.26)	-0.23 (-0.50, 0.14)
BSI-18 Somatization	0.03 (-0.28, 0.35)	0.15 (-0.17, 0.48)	-0.33 (-0.75, 0.09)	**-0.48 (-0.86, -0.11)**

Note: ImPACT Verbal missing 236, ImPACT Visual missing 238, ImPACT Visual Motor missing 237, ImPACT Reaction Time missing 236, BESS Total Score missing 192, SCAT Number of Symptoms missing 21, SCAT Symptom Severity missing 21, SAC Total Score missing 164, BSI-18 GSI Composite missing 31, BSI-18 Depression missing 31, BSI-18 Anxiety missing 31, BSI-18 Somatization missing 31.

Model adjusted for incident concussion, years of contact sports at baseline, time between baseline and exit evaluations, race, concussion at baseline, and primary position.

Supplemental Table 5. Weighted Adjusted Change in Concussion Battery Outcomes from Initial Baseline to Exit Assessment, Adjusting for Number of Prior Concussions (0, 1, 2+) Instead of for History of Prior Concussion (yes, no).

Outcome	Change in Overall Population	Change in No Incident Concussion Group	Change in Incident Concussion Group	Difference in the Change from Baseline to Exit Comparing Individuals with Versus without an Incident Concussion
ImPACT Verbal	0.79 (-1.42, 3.01)	0.67 (-1.68, 3.01)	1.44 (-2.15, 5.03)	0.78 (-2.87, 4.42)
ImPACT Visual	**4.59 (1.74, 7.43)**	**4.01 (1.08, 6.94)**	**7.44 (3.51, 11.36)**	3.43 (-0.02, 6.87)
ImPACT Visual Motor	**2.90 (1.78, 4.02)**	**2.88 (1.68, 4.07)**	**3.01 (1.62, 4.41)**	0.14 (-1.20, 1.48)
ImPACT Reaction Time	-0.01 (-0.03, 0.01)	0.00 (-0.03, 0.02)	-0.03 (-0.07, 0.00)	-0.03 (-0.06, 0.00)
ImPACT Symptom Severity	**1.50 (0.31, 2.70)**	**1.93 (0.68, 3.18)**	-0.66 (-2.49, 1.17)	**-2.59 (-4.38, -0.80)**
BESS Total Score	**-2.49 (-3.75, -1.23)**	**-2.61 (-3.93, -1.28)**	**-1.85 (-3.53, -0.17)**	0.76 (-0.86, 2.37)
SCAT Number of Symptoms	0.40 (-0.18, 0.99)	0.60 (-0.01, 1.20)	-0.63 (-1.34, 0.08)	**-1.22 (-1.88, -0.56)**
SCAT Symptom Severity	0.64 (-0.58, 1.86)	1.03 (-0.24, 2.30)	-1.46 (-3.03, 0.12)	**-2.49 (-4.03, -0.95)**
SAC Total Score	**0.08 (-0.33, 0.49)**	0.00 (-0.42, 0.42)	0.51 (-0.12, 1.14)	0.51 (-0.05, 1.07)
BSI-18 GSI Composite	0.25 (-0.44, 0.94)	0.41 (-0.33, 1.15)	-0.68 (-1.57, 0.22)	**-1.09 (-2.04, -0.14)**
BSI-18 Depression	0.16 (-0.15, 0.46)	0.20 (-0.12, 0.52)	-0.09 (-0.54, 0.36)	-0.29 (-0.74, 0.16)
BSI-18 Anxiety	0.05 (-0.22, 0.32)	0.09 (-0.20, 0.37)	-0.15 (-0.49, 0.19)	-0.24 (-0.57, 0.09)
BSI-18 Somatization	0.04 (-0.24, 0.32)	0.13 (-0.17, 0.42)	**-0.44 (-0.80, -0.07)**	**-0.56 (-0.94, -0.18)**

Note: Data are presented as mean differences (95% confidence intervals). Statistically significant changes are noted in bold. Model adjusted for years of contact sports at baseline, race, time between baseline and exit assessments, numbers of prior concussions at baseline (0, 1, 2+), and primary position.
